# A Strategy for Single-Run Sequencing of the Water Buffalo Genome: (I) the Use of Third-Generation Technology to Quickly Produce Long, High-Quality Reads

**DOI:** 10.3390/ani15202991

**Published:** 2025-10-15

**Authors:** Federica Di Maggio, Marcella Nunziato, Elvira Toscano, Leandra Sepe, Roberta Cimmino, Emanuela Antonella Capolongo, Alessandra Vasco, Giovanni Paolella, Francesco Salvatore

**Affiliations:** 1 CEINGE—Biotecnologie Avanzate “Franco Salvatore”, Via Gaetano Salvatore 486, 80145 Napoli, Italy; dimaggio@ceinge.unina.it (F.D.M.); nunziato@ceinge.unina.it (M.N.); toscano@ceinge.unina.it (E.T.); leandra.sepe@unina.it (L.S.); capolongo@ceinge.unina.it (E.A.C.); vasco@ceinge.unina.it (A.V.); 2 Dipartimento di Medicina Molecolare e Biotecnologie Mediche, Università degli Studi di Napoli Federico II, Via Sergio Pansini 5, 80131 Napoli, Italy; 3 Associazione Nazionale Allevatori Specie Bufalina (ANASB), 81100 Caserta, Italy; r.cimmino@anasb.it

**Keywords:** whole-genome sequencing, third-generation sequencing, long-reads, sequencing, water buffalo genome

## Abstract

**Simple Summary:**

The study of farm-bred animal genomes is crucial for enhancing their productivity, particularly in meat and dairy production. The mass of information resulting from these studies is potentially very useful in increasing the value of these animals by optimizing their breeding conditions, nutrition, and environmental living situations. A comprehensive genomic analysis, using both ‘typical’ and ‘new’ sequencing strategies, provides valuable insights for improving breeding conditions. Herein, we propose the use of a long-read sequencing strategy to obtain high-quality de-novo genome assembly. This technical note focuses on the methodologies and technologies used for sequencing the complete genome of five water buffaloes. We used long reads in the sequencing phase in order to achieve, in a short time and with limited effort, good coverage and assembly quality, possibly comparable to the most robust ones for this animal species.

**Abstract:**

(1) Background: Water buffaloes (*Bubalus bubalis*) are important for dairy and meat production. Up to now, genomic analysis has focused on female subjects, leaving the Y chromosome essentially unknown. Advances in third-generation sequencing (TGS) made it possible to improve the study of complex genome sequences, such as buffalo and other mammalian species including humans. (2) Methods: In this study, we applied TGS-based long-read sequencing to generate, in one step, high-quality whole-genome sequences, which can take full advantage of a rapid bioinformatic pipeline, such as that described in the companion paper. (3) Results: Five male buffalo genomes have been fully sequenced at relatively high depth (20–40×) which, combined with the read length typical of TGS, provide the basis for important insights into male-specific genetic traits, including those linked to meat and milk production. (4) Conclusions: With the use of TGS technologies, we offer a complete strategy for fast, one-step genome sequencing which can also be applied to other farm animals with a comparably large genome. This approach can help in revealing genetic features characteristic of an animal individual beyond the simple assessment of a number of SNPs or other known sequence variations, thus supporting improved genetic selection for dairy productivity and future research on genetic variability in buffalo breeds.

## 1. Introduction

In recent years, scientific progress in nucleic-acid sequencing has increased our knowledge of human and other animal genomes [1,2]. Since the start of the Human Genome Project and the publication of the first version of the human genome reference in the 2000s [3,4], the complete genome sequence of various animal and plant species (i.e., chickens, goats, dogs, cats, and yaks) have been analyzed and published [5,6,7,8,9]. In 2017 the first sequence of a female water buffalo was published [10]. Water buffaloes are farm-breeding animals that contribute to food production, meat, and milk. They are bred in different geographic areas, including India and the Mediterranean countries, where agricultural and environmental conditions have shaped their adaptation and productivity [11,12,13]. In this context, the knowledge of buffalo genome sequences appears of great interest for improving the assessment and quality, particularly of dairy products [10,11,12,14,15,16]. There are two subspecies of domesticated water buffalo that differ in geographic distribution, habitat, usage, and certain genetic characteristics [11,12,14]. The swamp buffalo, with 48 chromosomes (2N = 48), originates from Southeast Asia and is well-suited to swampy and muddy environments [14]. It is primarily used as a working animal in agriculture, and is valued for its strength and endurance. The river buffalo has 50 chromosomes (2N = 50), originates from South Asia [14] and is widespread in the Middle East and in parts of Europe. It adapts to drier, more open areas, and is a significant source of milk and cheese production.

Obtaining the complete genome sequence of water buffaloes was crucial for both basic and applied biology [10,15]. Research aimed at assembling the buffalo genome started several years ago. A first draft of the buffalo genome was published in 2017 and was obtained using short-read technologies on a female animal [10]. A significant advance was made in 2019 with a chromosome-level assembly, which is still the most comprehensive reference available. In the study by Wai Yee Low et al., genome assembly of a female Mediterranean buffalo was carried out using a combination of sequencing techniques to obtain a significantly more accurate genome than the first published one [10,16]. The study highlighted the advantages of using advanced, state-of-the-art technologies such as third-generation sequencing to overcome the limitations of NGS methodologies [16,17,18,19].

In recent years, third-generation sequencing strategies, provided by companies such as PacBio and Oxford Nanopore [20,21], have made significant contributions, particularly in reconstructing de-novo assemblies [22,23]. One of the main advantages of these technologies is the ability to obtain long reads, allowing the sequencing of single-stranded DNA regions up to 100 kb, much larger than the 400–500 bp typical of second-generation sequencing [17].

In this technical note, we discuss the use of TGS to quickly produce high-depth and -quality genome sequences to be used to study the genome of individual farm animals, searching for variations, and which can also be used for individual genome assembly.

## 2. Materials and Methods

### 2.1. Sample Selection

The samples used in this study were taken from the peripheral blood of male (*n* = 3) and female (*n* = 2) buffaloes of the Mediterranean breed and exclusively from the Campania region. The selection of the samples was carried out to ensure that samples were taken from individuals who were not related to each other. Herein, we describe the entire process used for 5 buffaloes. Blood samples were collected in EDTA-containing tubes, with two 5 mL tubes taken for each sample. The samples were subsequently transported on ice to the laboratory and processed within 2 h.

### 2.2. Nucleic Acid Extraction

We optimized a specific protocol for nucleic acid extraction [24,25] to obtain high-molecular-weight genomic DNA. This step is extremely delicate and crucial, as maximizing the length of DNA fragments is important. The length of the fragments was then selected during the preparation of the libraries, a crucial step in our workflow. In our case, obtaining long reads during the sequencing phase was essential, as they are critical for the de-novo assembly of the buffalo genome.

**Figure 1:**
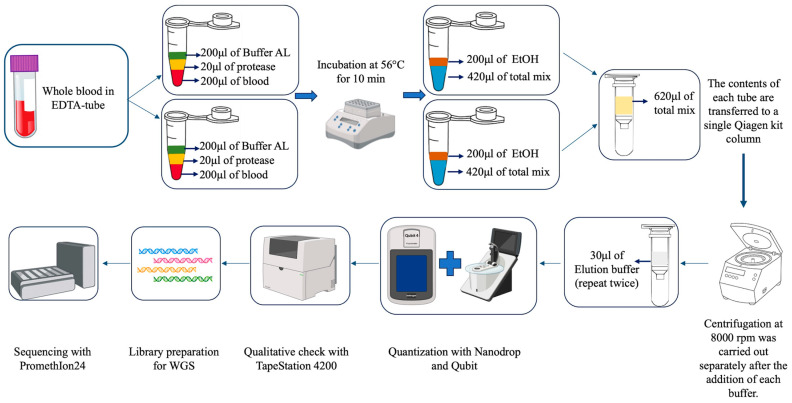
Flowchart of the extraction process of high-molecular-weight DNA from buffalo blood, followed by the complete workflow leading to sequencing via third-generation sequencers.

We utilized the QIAamp DNA Blood Mini Kit (Qiagen, Hilden, Germany, Cat. No. 51104) to extract DNA from blood samples, with some modifications to increase the DNA yield. Indeed, the use of the standard protocol had not allowed us to obtain the same yields that we were able to achieve with these modifications. This was because the starting blood was not of optimal quality, as the samples were taken without any sedation of the animals. The use of the double amount of blood therefore allowed us to obtain gDNA with a higher quantitative yield. Therefore, the variation introduced from the standard protocol proposed by the company consists of processing two times 200 µL (for a total of 400 µL) of blood separately in two distinct Eppendorf tubes, each with its own buffer. After incubation and washing with ethanol, the contents of the two tubes were combined in a single filter to proceed with the last steps of extraction. More in detail, in each tube we mixed 200 µL of buffalo blood with 20 μL of protease. Next, we added 200 μL of Buffer AL (lysis buffer) and incubated at 56 °C for 10 min. Then we added 200 μL of 100% ethanol in each tube (up to this step we have two tubes for each sample). The entire contents of each tube was transferred to a single column and centrifuged for 1 min at 8000 rpm (6010 rcf) using centrifuge 5424R Eppendorf. The process was continued according to the kit’s standard protocol. However, at the end of the process, rather than eluting the DNA in 200 μL of Buffer AE (containing 10 mM Tris-Cl and 0.5 mM EDTA; pH 9.0), we used 60 μL, performing the elution in two steps to maximize DNA recovery (see Figure 1).

At this point, we performed qualitative–quantitative checks via spectrophotometric and fluorometric techniques, such as Nanodrop and Qubit (Thermo Fisher Scientific, Waltham, MA, USA, version 3.0—Qubit dsDNA BR Assay Kit Cat. No. Q32850 and Qubit dsDNA HS Assay Kit Cat. No. Q32851), to assess the quality of the extracted high-molecular-weight DNA and the quantity obtained, expressed in ng/μL. We then performed further analysis via capillary electrophoresis using the TapeStation 4200 (Agilent Technologies, Santa Clara, CA, USA) with the Agilent TapeStation Controller software version 5.1 to verify the integrity of the DNA and determine the length of the fragments. The TapeStation is an automated electrophoresis system based on capillary microfluidics, which we have used for analyzing the quality and integrity of genomic DNA (gDNA). This system uses screen tapes pre-loaded with polymer gels and, depending on the type of sample to be tested, specific assays can be used; in our case we used the Genomic DNA ScreenTape assay (Agilent Technologies, Genomic DNA reagents Cat. No. 5067-5366 and Genomic DNA ScreenTape Cat. No. 5067-5365). The system uses a combination of electrophoresis and fluorescence to generate migration profiles and calculate the DNA integrity number (DIN). The DIN assesses the fragmentation of a genomic DNA sample by evaluating the signal distribution in the size range and assigning an automatically calculated score (DIN 10 indicates an intact sample whereas DIN 1 corresponds to very degraded DNA). At the end of this process, the DNA was stored at a controlled temperature of −20 °C.

### 2.3. Library Preparation and Long-Reads Sequencing

The first step involved confirming that the extracted genomic DNA (gDNA) was of high molecular weight. The check performed using the TapeStation 4200 (Agilent Technologies) indicated an average fragment length of over 60 kbases. Once the quality of the sample was established, the DNA was quantified using Qubit 3.0 and then diluted to a concentration of approximately 25 ng/µL in a final volume of 60 µL. Subsequently, fragmentation was performed using the Covaris g-TUBEs (Covaris Cat. No. 520079) to obtain DNA fragments between 20 and 30 kbases. Moreover, to verify the fragmentation, the samples were analyzed once again with the TapeStation 4200. After confirming that the fragments obtained were within the target range, we proceeded with the library preparation phase. At this point, library preparation was performed using the Ligation Sequencing Kit V10 and V14 (Oxford Nanopore Cat. No. SQK-LSK110 and SQK-LSK114), following the protocol provided by the manufacturer.

The damaged ends of each sample were repaired and reduced via the NEBNext^®^ Companion Module kit (New England Biolabs, Ipswich, MA, USA, Cat. No. E7180S). The repaired DNA was purified via Agencourt AMPure XP magnetic beads (Beckman Coulter, Brea, CA, USA, Cat. No. A63880) and eluted in 30 µL of water. At this point, the specific adapters for sequencing with Nanopore were added, which was essential for the next sequencing step. After the adapters were added, the DNA was purified with magnetic beads and eluted in elution buffer (EB). At all stages of library preparation, we took special care to avoid vortexing and not to perform steps involving excessive pipetting of the gDNA to prevent unwanted fragmentation. The main goal of this strategy was to obtain long sequences.

The flow cells used (Oxford Nanopore, Oxford, UK, Cat. No. FLO-PRO110 and FLO-PRO114M) were subjected to rigorous quality control following the standards laid down by Nanopore. Only flow cells with an available pore count of more than 5000 were used, as also indicated by the manufacturer. Once the flow cell has fewer than 5000 pores available for sequencing, the sequencer software warns the user that the flow cell is not suitable for use. For each flow cell, between 10 and 60 fmol of the library were loaded, calculated according to the length of the starting gDNA fragments. See Figure 1 for the complete workflow of the procedure from the original sample to Nanopore sequencing.

### 2.4. Long-Read Data Generation

**Figure 2:**
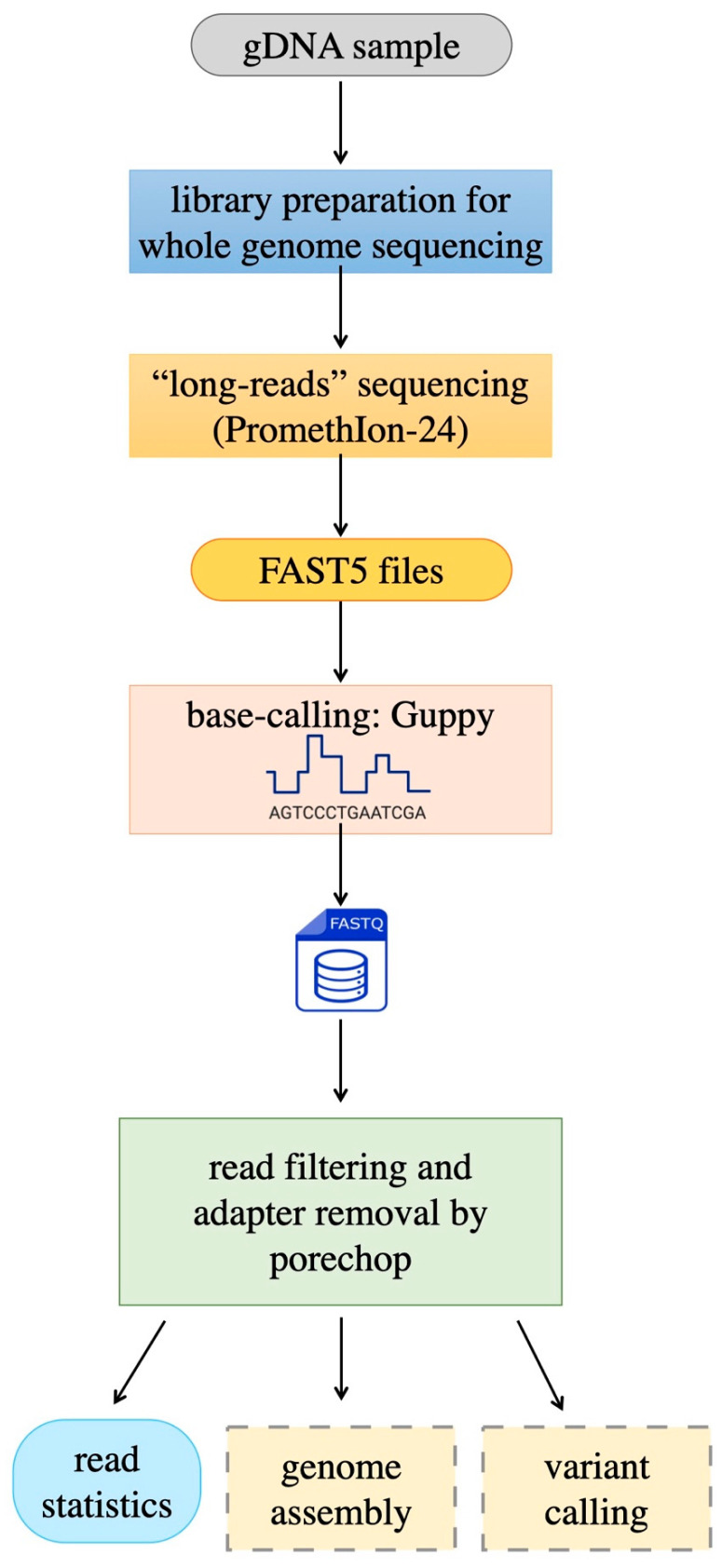
The sequencing workflow begins with library preparation; whole-genome sequencing is performed by means of a third-generation procedure (Oxford Nanopore) which generates raw FAST5 files. Basecalling produces FastQ files, which are, in turn, subjected to read preprocessing and quality evaluation. Sequences produced in this way are ready to be used for genome assembly, variant calling, or other uses, through procedures like those described in companion paper [30].

At the end of each run, PromethIon24 continuously generates a large amount of data. During this process, when DNA passes through the pores in the flow cells, a signal corresponding to the passage of 4–5 bases is detected [23]. This signal is produced by the variation in the ionic flow in the nanopore, generating valuable information. These raw data are stored in FAST5 files, a format based on Hierarchical Data Format 5 (HDF5) with a specific schema defined by Oxford Nanopore Technologies (ONTs). The FAST5 files contain all the information related to the run, including the current signal readings (see Figure 2).

Subsequently, the basecalling process transforms the raw signals into sequences, producing files in FASTQ formats. ONTs’ basecalling algorithms are based on neural networks, thereby ensuring high accuracy in decoding DNA sequences.

After 80 h, the time we decided on for sequencing, a final report was generated that provided all the information about the run. This report allows us to assess the final yield, which, for runs performed on PromethIon24 sequencers, is theoretically around 13 Tb of data.

### 2.5. Read Preprocessing and Quality Evaluation

The output of the sequencing procedure was evaluated using either of the basecalling tools proposed by the provider (Guppy 6.3.9, Dorado 7.1.4 and 7.2), each with the suggested run options, by using the default setting and the “super accurate” method. The use of different basecallers was due to the ongoing updates released by the manufacturer Nanopore, aimed at improving the accuracy of basecalling. As the runs were carried out at different times, it was necessary to adapt the sequencer software to the latest versions to ensure proper performance. In the basecalling procedure, reads with Phred quality scores lower than 10 (fail reads) were discarded, while the remaining ones were used for further analyses. A further preprocessing procedure by porechop (version 0.2.4) [26] was used to trim adapter sequences at the two ends of each read and to filter out chimeric reads (using the option--discard_middle), occasionally produced by ligation of the same adaptor to two different DNA fragments, using the following command:

porechop --i */path/to/input/directory/*--o */path/to/output/file.fastq.gz*--discard_middle

Statistical analysis and plot production was performed using R, either within the R Studio (1.4.1106 version) environment or directly accessed from within PHP scripts as previously described [27,28]. Further statistics were obtained by using Nanoplot (version 1.33.1) [29] to evaluate the final reads sets in terms of number, length, and average quality, using the following command:

NanoPlot --fastq_rich */path/to/input/file.fastq* --format pdf—maxlength 200000 --outdir */path/to/output/directory*

## 3. Results

### 3.1. Evaluation of Extracted DNA

High-molecular-weight DNA was extracted from blood samples of five individual buffaloes by using the procedures reported in Section 2. Quality and purity of each sample were assessed through qualitative and quantitative analyses, including absorbance evaluation at 260, 280, and 230 nm; 260/280 and 260/230 ratios were used to check for contaminants such as proteins, phenol, or other extraction residues. The process, performed on all five samples, resulted in DNA concentrations between 67 and 202 ng/µL, corresponding to about 4–12 µg total of sample. The 260/280 and 260/230 ratios were always between 1.5 and 2 for all the samples, except for one (b_3, in which 260/280 was 1.7 and 260/230 was 1.1); however, the latter produced sequences not different from the others (see below), showing that genome sequencing was achievable even in the presence of suboptimal sample purity indices and that, therefore, the strategy remains applicable even when starting from blood samples with quality metrics below standard threshold values.

Samples were further characterized by analyzing them with a Agilent TapeStation 4200 to determine the DNA integrity number (DIN, ranged from 1 to 10 where higher number indicates higher DNA integrity and, of course, very low degradation). DIN values were always very close to approximately nine for the whole set of samples, indicative of a good consistency in the results obtained from blood samples (see Table 1).

### 3.2. Long-Read Sequencing

The complete procedure is schematically reported in Figure 2. DNA was fragmented to an average size of 20–30 kbases; size was checked, again by running samples on Agilent TapeStation 4200 (see Section 2). Library preparation for long-read sequencing was performed by using the ligation sequencing kits provided by Oxford Nanopore (see Section 2). Sequencing was performed on Promethion from the same company, using a long-read strategy. At the end of each run, which typically lasted 80 h, the raw data were collected, stored, and converted into reads through a basecalling step.

The basecalling procedure relied on two different tools, both suggested by the provider, *Guppy* and *Dorado*, with the latter in two different versions. In this phase, we assessed how many reads were accepted after discarding low-quality ones: typically, using the “super accurate” method for basecalling, i.e., a quality threshold of 10 for MinQ (average Phred quality score), over 75% passed the test while approximately 20–25% of the generated reads did not pass the quality filters and were classified as failed reads (see Table 2).

While preparing libraries, two different kits, SQK-LSK110 and SQK-LSK114 (Table 2), were used to match two types of flow cells, corresponding to the respective kits (R.9 for SQK-LSK110 and R.10 for SQK-LSK114). The two kits were supposed to substantially differ in terms of performance, chemistry compatibility, and consistency. SQK-LSK110 offers a faster, more straightforward workflow with generally consistent results across different samples, even when DNA-input quality is suboptimal, at the expense of read accuracy which typically remains moderate (Q10–Q15). SQK-LSK114 was optimized for the newer R10.4.1 flow cells, though being more sensitive to input-DNA quality and execution of the protocol. In our hands, samples prepared with the two kits produced similar values, ranging between 99 and 135 Gbases produced in a single run. Similarly, the number of reads was in the range of 9–15 million for the libraries prepared with both methods, with a read N50 between 11 and 17 kbases. To reduce the effect of variability in sample preparation, the two kits/procedures were used to build libraries and execute the sequencing step from the same DNA sample (b_1 and b_2); in both cases, the results, reported in Supplementary Table S1, did not show large differences between the two procedures, except for a moderately larger number of total bases for sample b_2 with kit SQK_LS114 (116.39 Gbases), associated with a major number of smaller sequences (11.62 millions).

### 3.3. Read Preprocessing and Quality Evaluation

The reads produced in the basecalling step were then processed to trim adapter sequences at the two ends of each of them and to filter out wrong reads, such as chimeric reads occasionally produced by ligation of the same adapter molecule to two different DNA fragments. This step was carried out by using *porechop* (version 0.2.4) [26], a program specifically designed for finding and removing adapters from Oxford Nanopore reads.

**Figure 3:**
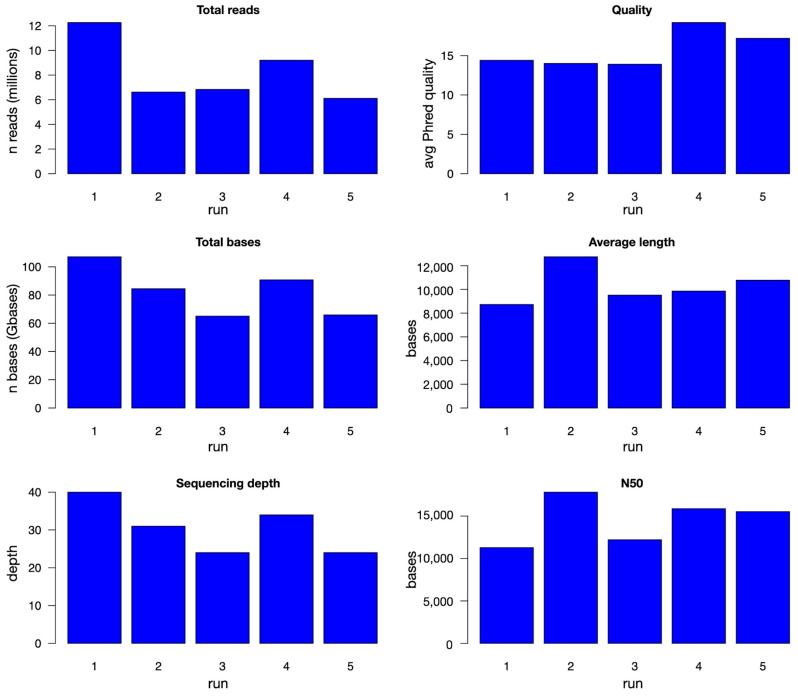
Quality evaluation of preprocessed reads. For each of the five runs, number of sequenced reads and bases, sequencing depth, average Phred quality score, average read length, and N50 are reported in bar plots.

Final evaluation of the sequences obtained after preprocessing and filtering is reported in Figure 3 and numerically in Supplementary Table S2. The number of final reads was between 6 and 12 million, for the five runs reported in Table 2, for a 65–107 Gbase range, corresponding to an expected depth of about 24–40×. The number of bases after filtering is almost identical to the number of bases of pass reads, with the only possible exception of sample b_2, an indication of the good performance of the library-building steps. Average Phred quality score was about 14 for the first three runs, corresponding to an error rate of about 4%, while the remaining two, sequenced with the library protocol SQK_LS114, showed even better values, up to about 19 (1.3% error rate). Average read length was between 8 and 13 kbases, with N50 between 11 and 18 kbases. The difference in average quality scores between sequences obtained with the two procedures is also observed when comparing the results of using the two protocols on the same DNA (see above and Supplementary Table S1). The other parameters do not show big differences between the two protocols, as previously observed for unprocessed reads.

**Figure 4:**
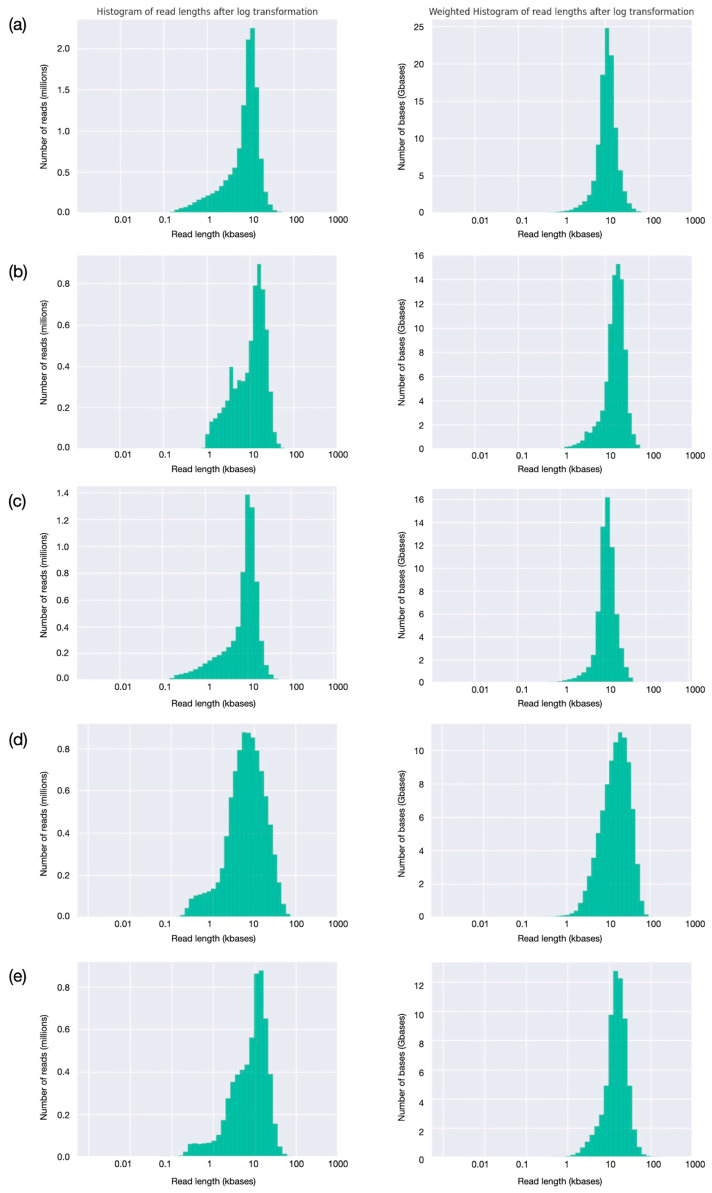
Evaluation of read-length distribution (**a**–**e**). For each of the five runs, corresponding to the five buffaloes, see Table 2, the histogram reports read-length distribution and total number of bases for a given read length, reported in the first and second columns, respectively.

**Figure 5:**
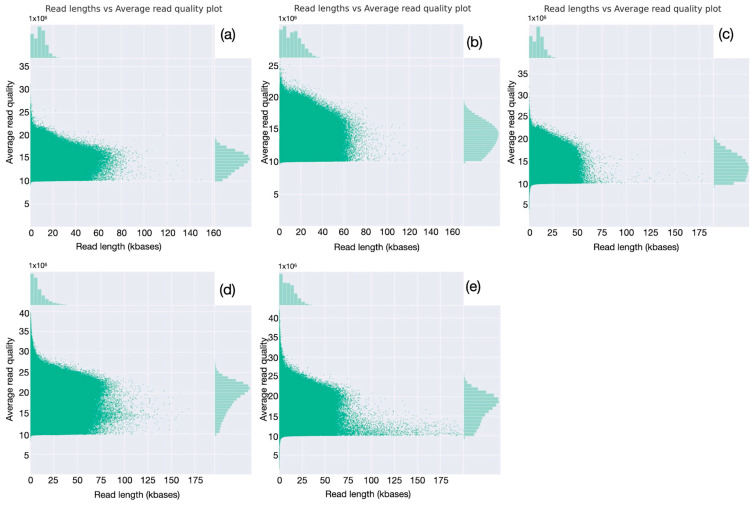
Evaluation of the distributions of read length vs. quality (**a**–**e**). For all five runs, buffalo numbers are in Table 2; for each read, quality is reported as a function of length; the respective distributions are reported on the corresponding axis.

Read analysis was extended by evaluating read-length distributions (Figure 4). All the runs have a relatively tight distribution, with the greatest number of reads having lengths close to the calculated mean (9–12 kbases). A very small fraction of reads are shorter, down to 1000 bases or less, while a few long reads are longer than 60–70 kbases (Figure 4, first column). For all the runs, the large majority of bases is provided by reads of lengths between 5 and 20 kbases (Figure 4, second column). Evaluation of read quality as a function of length (Figure 5) shows, for the first three runs, obtained with SQK_LS110 kit and with average Phred quality score around 14.0, that quality ranges between 10 and 25 for almost all the sequences; better-quality scores, up to 35, were observed only for the last two ones, produced using SQK_LS114 kit and with average Phred quality score around 19.0. As might be expected, for all the runs, the longer the read, the lower the quality score; however, quality well above average is observed for many sequences longer than the average read length, indicating that high read quality is well distributed and involves most of the produced sequences.

## 4. Discussion

The advent of new sequencing technologies and the significant progress made in this area enabled researchers to leverage these innovations for the study of human and animal biology [17,18,19], including genome-wide methylation analyses [2,18,31,32]. This technology can be used in several application areas, one of which is the creation of reference genomes. These genomes are important tools for various purposes, ranging from animal tracking to sustainable management and the qualitative and quantitative improvement of agri-food production. These genomic data also find direct applications in animal breeding and dairy farms, offering new tools to support genetic advancement, optimize production performance, and improve the health status of animals. Through such resources, farms can adopt more effective genetic-selection strategies, develop more sustainable production lines, and address food-quality and safety challenges. Currently, the importance of having a reference genome for a male buffalo individual is becoming increasingly clear. To date, sequencing efforts and the available reference genome in the literature refer only to a female individual for water buffaloes [16]. This limitation is a significant barrier to research, as understanding male-specific genetic traits may be important for many areas of study, such as evolutionary genetics, reproductive selection, and genetic improvement. Obtaining a complete genome sequence of a male specimen would fill this gap, thereby providing a more complete and detailed view of the genetic heritage of the species. In this context, state-of-the-art technologies are playing a central role. The adoption of standardized molecular biology protocols represents a further step toward greater efficiency in the production of whole-genome sequences. These protocols ensure the quality and reproducibility of the data, thereby improving their value for both scientific research and industrial applications. In particular, the use of advanced technologies capable of generating long genome sequences is becoming increasingly important. Such sequences are useful in the assembly steps of reference genomes as they make it possible to overcome the limitations of short-read technologies, since they result in a minor number of contigs, reduce the risk of fragmentation, and improve the accuracy of the final assembly. Overall, advances in genomic sequencing, combined with the integration between innovative technologies and standardized protocols, are contributing to developments in animal biology and may have positive implications for related production sectors.

The aim of this study was to present, though on few animals, a novel strategy based on long-read sequencing in order to enable the reconstruction of even large genomes within a few days. In the protocol described in this technical note, we show how high-molecular-weight and high-quality DNA can be obtained. High-molecular-weight and high-quality DNA are essential to ensure the success of the subsequent steps, such as the preparation of libraries for WGS and third-generation sequencing. There is a correlation between DNA purity and integrity and the reliability of long-read sequencing, as showed herein. During our pilot study, we also compared the performance of two different kits (SQK-LSK110 and SQK-LSK114) which were initially proposed by the same manufacturer. The results showed no significant differences in the amount of data generated, suggesting that both strategies are equivalent from the point of view of overall yield.

The strategy discussed in this study concerns long-read sequencing using Oxford Nanopore technology in order to obtain a de-novo assembly of the buffalo genome, as discussed in the companion paper [30]. Despite the significant technological advances made to improve the error rate of nanopore sequencing, some inherent limitations related to base-reading accuracy remain. However, the impact of sequencing errors may be minimized by performing simultaneous runs of the same sample. This approach would not only improve overall data quality but also increase coverage, further reducing the overall error compared to a single run.

The whole strategy, from the collection of blood to the production of long-reads by simplifying the procedures, can accelerate the assembly of individual genomes, including, as in this case, sequences from male subjects and allowing further knowledge about the sequence of male-sex chromosomes in buffaloes. Therefore, we generated long-read datasets and validated library and sequencing workflows sufficient to support de-novo assembly; assembly is being reported separately (companion paper) [30].

## 5. Conclusions

In conclusion, we implemented a complete sequence strategy using the most recent methodology of sequencing nucleic acids (third-generation sequencing by long reads) and the most recent data analysis methodologies.

The choice of a male river buffalo was significant, as its genome had not been previously sequenced to this extent. Our results provide a valuable basis for the subsequent evaluation of farm-breeding animal productivity, particularly for milk and dairy products. The data obtained may be a starting point for improving genetic selection programs and to increase the productivity of farm animals, particularly for the production of milk and dairy products. Furthermore, this work offers opportunities for future perspectives, such as the development of genetic markers and especially the scaling-up to other buffalo populations or breeds to assess their genetic variability. A limitation of this approach is, of course, the depth achievable in a single run, which necessarily constrains the quality of the results to be produced in following steps such as variant search or genome assembly. Additionally, while Oxford Nanopore sequencing is known to have a slightly higher error rate compared to other platforms, its ability to generate long single-strand reads—often spanning 10 to 15 kbases pairs—remains a major advantage. This long-read capacity is invaluable when attempting genome reconstruction, particularly in complex or repetitive regions, and, in most cases, largely compensates for the higher base-level error rate.

## Figures and Tables

**Table 1:** All the information of the DNA extractions performed on the 5 analyzed samples.

N°	ID_Buffalo	Sex	Concentration (ng/ µL)	DIN
1	b_1	M	113.7	9.1
2	b_2	M	133.3	9.1
3	b_3	F	156.3	9.3
4	b_4	M	67.9	9.0
5	b_5	F	202	8.8

**Table 2:** Information on each run performed for whole-genome sequencing, including the raw data obtained for each run.

				Long-Reads Sequencing Details							
N°	ID Buffalo	Uploaded Library (fmol)	Flow Cell ID	Initial Pores	Library Protocol (WGS)	Basecaller Used	Estimated Bases (Gbases)	N50 (kb)	Number of Reads (M)	Bases Called (Mbases)	
										Pass	Fail
1	b_1	58	PAM72151	6175	SQK-LSK110	Guppy 6.3.9	135.66	11.27	15.52	107.99	21.72
2	b_2	33	PAM31376	6734	SQK-LSK110	Guppy 6.3.9	107.13	17.77	8.51	90.1	15.91
3	b_3	32	PAI33833	9177	SQK_LSK110	Dorado 7.1.4	106.64	12.18	11.78	65.37	41.64
4	b_4	11	PAI27564	5180	SQK-LSK114	Guppy 6.3.9	111.69	15.71	11.03	91.14	16.26
5	b_5	36	PAU60985	7066	SQK-LSK114	Dorado 7.2	99.35	15.44	8.84	66.14	27.45

ID_buffalo: identifying number. Uploaded library (fmol): quantity in fmol of DNA library loaded into each flow cell. Flow cell ID: Identification number of each flow cell used. Initial Pores: number of pores available at the time of the flow-cell check. Library protocol (WGS): type of kit used for library preparation. Basecaller used: type of basecaller used. Estimated bases: number of bases sequenced in a single run in Gbases. N50: a value (expressed in kbases) where half of the data obtained is contained within reads with alignable lengths greater than that. Number of Reads: number of reads generated in a single run expressed in millions. Bases called pass (those with Phred quality scores higher than 10): number of bases that passed basecalling filters. Bases called fail: number of bases that did not pass basecalling filters.

## Data Availability

Sequencing-related data, including raw reads, are available at the following address: http://genobu.ceinge.unina.it/bufreads/ (accessed on 2 October 2025). Any further data related to the article will be made available by the authors on request.

## Supplementary Materials

The following supporting information can be downloaded at: https://www.mdpi.com/article/10.3390/ani15202991/s1, Table S1: Comparison of chemistry used in two different buffaloes. Table S2: Read quality evaluation per sample after filtering.

## Abbreviations

The following abbreviations are used in this manuscript:

CNV	Copy number variant
gDNA	Genomic DNA
HDF5	Hierarchical Data Format 5
ONT	Oxford Nanopore Technologies
ssDNA	Single-strand DNA
dsDNA	Double-strand DNA
DIN	DNA Integrity Number
WGS	Whole-genome sequencing
TGS	Third-generation sequencing
