# Supplementary material for: A Strategy for Single-Run Sequencing of the Water Buffalo Genome: (I) the Use of Third-Generation Technology to Quickly Produce Long, High-Quality Reads
Source: Animals (Basel). 2025 Oct 15;15(20):2991. doi: 10.3390/ani15202991 (PMC12560924; doi:10.3390/ani15202991)
Supplement: Supplementary file 1 [file animals-15-02991-s001.zip › animals-3819686-supplementary.pdf]

Technical Note

A Strategy for Single-Run Sequencing of the Water Buffalo Genome: (I) the Use of Third-Generation Technology to Quickly Produce Long, High-Quality Reads

Federica Di Maggio [1,2,†], Marcella Nunziato [1,2,†], Elvira Toscano [1,†], Leandra Sepe [1,2], Roberta Cimmino [3], Emanuela Antonella Capolongo [1], Alessandra Vasco [1], Giovanni Paolella [1,2,*] and Francesco Salvatore [1,2,*]

[1] CEINGE—Biotecnologie Avanzate "Franco Salvatore", Via Gaetano Salvatore 486, 80145 Napoli, Italy; dimaggio@ceinge.unina.it (F.D.M.); nunziato@ceinge.unina.it (M.N.); toscano@ceinge.unina.it (E.T.); leandra.sepe@unina.it (L.S.); capolongo@ceinge.unina.it (E.A.C.); vasco@ceinge.unina.it (A.V.)

[2] Dipartimento di Medicina Molecolare e Biotecnologie Mediche, Università degli Studi di Napoli Federico II, Via Sergio Pansini 5, 80131 Napoli, Italy

[3] Associazione Nazionale Allevatori Specie Bufalina (ANASB), 81100 Caserta, Italy; r.cimmino@anasb.it

* Correspondence: giovanni.paolella@unina.it (G.P.); salvator@unina.it (F.S.); Tel.: +39-0817463019 (G.P.); +39-0813737826 (F.S.)

† These authors contributed equally to this work and should be considered co-first authors.

Table S1. Comparison of chemistry used in two different buffaloes.

		reads passed after base-calling step		reads after filtering and removal of adapter sequences					
Buf	kit	total bases (Gbases)	number of reads	total bases (Gbases)	number of reads	mean length	N50	mean quality	exp depth
b_1	SQK_LS110	107.99	12,295,453	107.13	12,269,837	8,731.0	11,283	14.4	40x
	SQK_LS114	117.05	13,154,403	116.52	13,153,569	8,858.1	10,865	19.1	43x
b_2	SQK_LS110	90.10	6,632,382	84.48	6,624,578	12,752.6	17,814	14.0	31x
	SQK_LS114	116.39	11,618,477	115.90	11,617,818	9,976.2	11,551	19.0	43x

Table S2. Read quality evaluation per sample after filtering.

		b_1	b_2	b_3	b_4	b_5
total bases raw (Gbases)		135.66	107.13	106.64	111.69	99.35
total bases pass (Gbases)		107.99	90.10	65.37	91.14	66.14
Filtered	total bases (Gbases)	107.13	84.48	65.08	90.77	65.94
	number of reads	12,269,837	6,624,578	6,838,190	9,207,831	6,117,981
	mean length (bases)	8,731.0	12,752.6	9,516.5	9,858	10,777.4
	N50 (bases)	11,283	17,814	12,208	15,869	15,519
	mean quality	14.4	14.0	13.9	19.2	17.2
	exp depth	40x	31x	24x	34x	24x
